# Typology of changes in quality of life over 12 months among currently or formerly homeless individuals using different housing services in Quebec, Canada

**DOI:** 10.1186/s12955-021-01768-y

**Published:** 2021-04-21

**Authors:** Gesthika Kaltsidis, Guy Grenier, Zhirong Cao, Nadia L’Espérance, Marie-Josée Fleury

**Affiliations:** 1 Department of Psychiatry, McGill University, Montreal, QC Canada; 2 Douglas Hospital Research Centre, Douglas Mental Health University Institute, 6875 LaSalle Blvd., Montreal, QC H4H 1R3 Canada; 3 Centre Intégré Universitaire de Santé et de Services Sociaux, Trois-Rivières, Quebec Canada

**Keywords:** Homelessness, Quality of life, Housing status, Factors, Typology, Cluster analysis

## Abstract

**Background:**

In health and social service evaluations, including research on homelessness, quality of Life (QOL) is often used as a key indicator of well-being among service users. However, no typology has been developed on changes in QOL over a 12-month period for a heterogenous sample of homeless individuals.

**Methods:**

Cluster analysis was employed to identify a typology of change in QOL for 270 currently or formerly homeless individuals using emergency shelters, temporary housing (TH) and permanent housing (PH) services in Quebec (Canada). Participant interviews were conducted at baseline and 12 months later. An adapted Gelberg–Andersen Model helped organize QOL-related sociodemographic, clinical, and service use variables into predisposing, needs, and enabling factors, respectively. Comparison analyses were performed to determine group differences.

**Results:**

Four groups emerged from the analyses: (1) young women in stable-PH or improved housing status with moderately high needs and specialized ambulatory care service use, with improved QOL over 12 months; (2) middle-age to older men with stable housing status, few needs and low acute care service use, with most improvement in QOL over 12 months; (3) older individuals residing in stable-PH or improved housing status with very high needs and reduced QOL over 12 months; and (4) men in stable-TH or worse housing status, with high substance use disorder, using few specialized ambulatory care services and showing decline in QOL over 12 months.

**Conclusions:**

Findings suggest that positive change in QOL over 12 months was mainly associated with fewer needs, and stability in housing status more than housing improvement. Specific recommendations, such as assertive community treatment and harm reduction programs, should be prioritized for individuals with high needs or poor housing status, and among those experiencing difficulties related to QOL, whereas individuals with more favourable profiles could be encouraged to maintain stable housing and use services proportional to their needs.

## Introduction

Quality of Life (QOL) is a principal outcome in health and social service evaluations used as an indicator of well-being among service users [1, 2]. Improving QOL is at the heart of the recovery paradigm which guides mental health (MH) care and interventions for homelessness [3, 4]. Research using QOL has primarily focused on patients with MH disorders (MHD) [5, 6], including those who experience homelessness [7, 8], especially veterans [9, 10] and newly admitted housing service users [11, 12].

A recent systematic review on the effectiveness of permanent subsidized housing and income assistance programs highlighted that QOL after 12 months was higher among formerly homeless individuals living in permanent housing (PH), including Housing First programs, compared to those receiving ‘treatment as usual’ [13]. Other research found that QOL increased with age among people who were currently or formerly homeless [8, 12, 14]; yet QOL was consistently lower among homeless individuals with serious and complex health problems, such as MHD, substance use disorders (SUD), or high functional disability [8, 15]. However, few studies have investigated QOL among homeless individuals using a range of short-term (emergency shelters), medium-term (temporary housing; TH) and long-term (PH) housing services [16–18]. Moreover, change or stability in QOL depends on various factors including change in housing status [19–21]. To our knowledge, no study to date has focused on change in QOL among homeless individuals based on their housing trajectories.

Typological research may be useful in identifying common factors related to change in QOL for a heterogenous group of homeless individuals using a variety of housing services. Previous typologies have classified homeless samples in terms of housing outcomes [22–24], patterns of emergency shelter use [25], previous life experience [26, 27], and physical or MH issues [23, 28, 29]. Typological research on QOL in homelessness has mainly involved cross sectional studies using baseline measures of participant QOL. [18, 30]. Therefore, identifying profiles of homeless individuals based on change in QOL over time would provide a novel contribution to understanding perceived well-being among diverse housing service users.

Cluster analyses of homeless individuals have considered multiple variables, including sociodemographic (e.g. age, sex), clinical (e.g. MHD, SUD) and health service use (e.g. emergency department [ED] visits, hospitalizations) variables [22, 23, 26, 31]. Yet despite high prevalence of problems in health and social functioning within the homeless population [32], studies have rarely examined variables like perceived health, functional disability, and use of public and community-based services. Moreover, few typologies have been developed using a conceptual framework. One model relevant to health service evaluation is the Gelberg-Andersen Behavioral Model for Vulnerable Populations [33], which classifies sociodemographic variables as *predisposing factors*, clinical variables as *needs*, and service-related variables as *enabling factors*. Previous homelessness research using the Gelberg–Andersen Model has focused on exit from supported housing [34], health service use [35] and service satisfaction [36]. However, no known cluster analysis exists to date on homelessness and QOL using the Gelberg–Andersen Model.

The objective of this study was to develop a typology of change in QOL at 12-month follow-up based on an adapted Gelberg–Andersen Model for a sample of 270 homeless individuals using different housing services in Quebec (Canada). Gaining insight on shared characteristics that influence QOL among subgroups of homeless individuals may contribute to knowledge, and guide housing policy and service improvements to better address needs in this population.

## Methods

### Study setting and data collection

This study was set in two major Canadian cities: Montreal and Quebec City. Prospective study participants were recruited from 27 community or public organizations, 20 providing housing services (five emergency shelters, 12 organization providing TH, and three offering PH), and seven organizations offering other essential services like food banks, day centers, soup kitchens, etc.

Eligible participants had to be at least 18 years old, with current or former experience of homelessness, and use one of the following housing services: PH (within the previous 2 years), TH (3–12-month residency), or emergency shelter. Recruitment by the project co-ordinator took place on-site, and housing staff made referrals after attending information meetings on the study. Posters were also displayed in common areas of the selected organizations inviting participant self-referral. While all interested individuals who met study eligibility criteria were included, the interviews for participants found intoxicated or otherwise unfit were delayed.

Interviews took place at the selected organizations, participant apartments, or local restaurants and were administered by trained research assistants. Baseline interviews (T0) were conducted between January and September 2017, generally within a day or shortly after initial contact with participants and were about 75 min in duration. The 12-month follow-up interviews took place throughout 2018 (T1), lasting only 55 min, as most sociodemographic data were collected at T0. Participants responded to questions on socio-demographics (e.g., age, education), and on clinical (e.g. MHD, perceived health), and service use (e.g., public primary care, community-based services) issues. Participants provided written, informed consent prior to the interviews. After completion of their interviews, participants were compensated for their time and contribution to the study. The study protocol was approved by the research ethics board of a MH university institute.

### Conceptual framework, variables and instruments

**Fig. 1:**
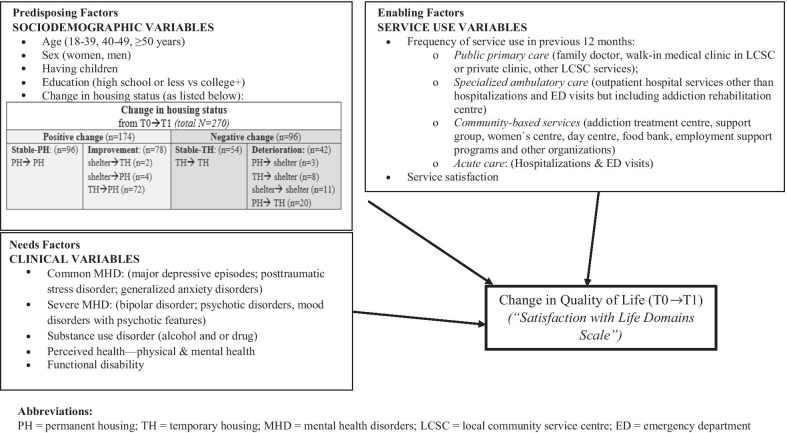
Conceptual framework for change in quality of life at 12 months (based on the Gelberg–Andersen Behavioral Model) [33]. Data collection at T0 (January-September 2017) and T1 (January-December 2018). Study variables organized into predisposing, needs, and enabling factors on the variable of interest, change in quality of life at 12 months (T1).

The variable of interest was “change in QOL from T0 (baseline) to T1 (12 months later)”. QOL was measured using the Satisfaction with Life Domains Scale (SLDS) (French version) [37], initially published by Baker and Intagliata [38]. The SLDS assesses 20 items on life satisfaction across five domains (daily life and social relations, housing and neighbourhood, personal relationships, spare-time activities, autonomy) with a 5-point Likert scale (1 to 5; higher = better QOL). Positive change in QOL over 12 months was represented as an increase in QOL score from T0 to T1, whereas a decreased score denoted negative change in QOL. The selection of independent variables was guided by the homelessness literature pertaining to QOL [12, 21, 39]. Figure 1 presents all the study variables organized into predisposing, needs and enabling factors according to the Gelberg–Andersen Model, while Table 1 lists the standardized instruments used [37, 40–45]**.** Predisposing factors included: age, sex, having children, education, and change in housing status over 12 months [with participants identified in one of the following four conditions: 1. deterioration (PH to TH or shelter, TH to shelter, or shelter to shelter by T1); 2. stable-TH (no change); 3. stable-PH (no change); and 4. improvement (shelter to TH or PH, or TH to PH)]*.* Needs factors for the previous 12 months included: common MHD (e.g., major depressive episodes, generalized anxiety disorders), severe MHD (e.g., bipolar disorder, psychotic disorders), SUD (alcohol and or drug), perceived health (both physically and mentally) and functional disability. Enabling factors were frequency of service use in the previous 12 months (public primary care, specialized ambulatory care, community-based services and acute care, including hospitalizations and ED visits) and overall service satisfaction score.

**Table 1:** List of all instruments used in study

Instrument	Variable(s) tested	Description	Psychometric properties
Satisfaction with life domains scale (SLDS) [37]	Quality of life (QOL)	20-item self-report scale on subjective QOL; across 5 domains (daily life and social relations, housing and neighbourhood, personal relationships, spare-time activities, autonomy); five-point scale (1 to 5); rating: 20 to 100 where higher = better QOL	Cronbach’s alpha = 0.92
Canadian community health survey (CCHS)—adapted [40]	Housing status	Three types of housing (emergency shelter, temporary housing, permanent housing with or without support)	N/A
	Age	Numerical value calculated from date of birth	
	Sex	Two-point scale (male = 1, female = 2)	
	Having children	Two-point scale (no = 0; yes = 1)	
	Education	Two-point scale (high school or less = 1, college or more = 2)	
	Perceived health	Two self-report questions rating current state of physical and mental health on five-point scale where 1 = poor to 5 = excellent	
M.I.N.I international neuropsychiatric interview 6.0 [41]	Mental health disorders (MHD)	120-item structured diagnostic interview for DSM-IV and ICD-10 psychiatric disorders; two-point scale (no = 0; yes = 1)	Kappa Cohen = 0.50–0.84
Alcohol use disorders identification test (AUDIT) [42]	Substance use disorder (alcohol)	10-item self-report scale to measure alcohol consumption; with zero to four-point scoring for multiple-choice questions; rating: 0–50 where higher = greater level of SUD for alcohol	Cronbach’s alpha = 0.74
Drug abuse screening test (DAST) [43]	Substance use disorder (drug)	28-item self-report scale used as a screening tool for drug consumption; two-point scale (no = 0; yes = 1); rating: 0–20 where higher = greater level of SUD for drugs	Cronbach’s alpha = 0.88
WHO disability assessment schedule 2.0 [44]	Functional disability	12-item short version assessment used for all diseases (physical illness and MHD); across 6 domains of functioning (cognitive, mobility, self-care, getting along, life activities, and participation); five-point scale (1 to 5); rating: 0 to 60 where 0 = no disability and 60 = full disability	Cronbach’s alpha = 0.93–0.94
Service utilization questionnaire (SUQ) adapted from CCHS [45]	Frequency of public primary care services use	*Public primary care* refer to family doctor, walk-in medical clinic in local community service centre (LCSC) or private clinic, other LCSC services; numerical value based on previous 12 months	N/A
	Frequency of specialized ambulatory care services use	*Specialized ambulatory care* refer to outpatient hospital services other than hospitalizations and emergency department visits but including addiction rehabilitation centre; numerical value based on previous 12 months	
	Frequency community-based services use	*Community-based services* refer to addiction treatment centre, support group, women’s centre, day centre, food bank, employment support programs and other organizations; numerical value based on previous 12 months	
	Frequency of acute care services use	*Acute care* refer to hospitalizations and emergency department visits; numerical value based on previous 12 months	
	Service satisfaction	Overall satisfaction with services; five-point scale (1 to 5) based on previous 12 months where higher = more satisfied with services	

### Analysis

Comparative analyses were conducted using chi-square tests on categorical variables (e.g. sex) and T-tests on continuous variables (e.g. age, functional disability) to test differences between baseline (T0) and 12 months later (T1). Missing values (less than 5%) were randomly distributed and imputed by the means [46, 47]. The k-means cluster algorithm [48, 49] with Gower dissimilarity coefficient [50] was used to identify subgroups of homeless individuals, based on change in QOL over 12 months and predisposing, needs, and enabling factors in the previous 12 months. To determine the optimal number of groups, several k-means solutions with different numbers of groups were computed for the cluster analysis. The four-group model was chosen based on the largest Calinski–Harabasz pseudo-F value, indicating that this was the most distinct solution for all the groups. To determine statistical differences between the groups, pairwise comparisons were conducted using the chi-square test or Fisher’s exact test for categorical variables, and T-tests or Wilcoxon rank-sum tests for continuous variables. Statistical analyses were performed using Stata 15.

## Results

At baseline (T0), of 497 individuals eligible for the study, 455 enrolled, including 45 shelter users, 229 TH and 181 PH residents. Twelve months later (T1), 270 participants were followed up, including 22 shelters users, 76 TH, and 172 PH residents, for a response rate of 59%. No significant differences were reported between T0 and T1 in comparative analyses for sex, age, or disability scores (sex: *p* = 0518 age: *p* = 0.126; disability score: *p* = 0.677). There were no significant differences in baseline characteristics for sex (*p* = 0.199), education (*p* = 0.689), and disability score (*p* = 0.330) between individuals retained (N = 270) or those lost to follow-up (N = 185).

The average baseline QOL score (71.05) improved by 2.1 (S.D. = 12.3) over 12 months, as shown in Table 2. Participants were mainly male (58%), ≥ 50 years old (57%) and 44% had at least one child. Regarding changes in housing status from T0 to T1, 16% of participants experienced deterioration in housing status, 20% remained in TH, 36% maintained PH and 29% improved their status. In terms of needs factors, the main diagnosis was common MHD (43%), followed by SUD (38%), and severe MHD (27%). The mean perceived state of health score was 7.1/10 (S.D. = 1.9), and functional disability score was 20.8/60 (S.D. = 7.3). Concerning enabling factors, over the 12-month study period, average frequency of public primary care use was 4.6 (S.D. = 7.8), specialized ambulatory care 2.5 (S.D. = 10.0), community-based services 90.2 (S.D. = 125.0), and acute care use was 2.5 (S.D. = 9.1). Satisfaction with services averaged 4.1/5 (S.D. = 0.8).

**Table 2:** Description of clusters based on change in QOL over 12 months according to variables included in the analysis and comparison between groups

	Group 1		Group 2		Group 3		Group 4		Total	
	n/mean	%/SD	n/mean	%/SD	n/mean	%/SD	n/mean	%/SD	n/mean	%/SD
Group size	57	21.11	87	32.22	66	24.44	60	22.22	270	100.00
Variable of interest										
Change in QOL over 12 months (mean/SD)^a^	4.60^3,4^	12.41	7.21^3,4^	10.78	− 3.17^1,2^	11.91	− 1.88^1,2^	11.32	2.10	12.32
Predisposing factors										
Change of housing status^b^										
Deterioration	0^2,4^	0.00	6^1,3,4^	6.9	0^2,4^	0.00	36^1,2,3^	60.00	42	15.56
Stable-TH	2	3.51	23	26.44	5	7.58	24	40.00	54	20.00
Stable-PH	24	42.11	39	44.83	33	50.00	0	0.00	96	35.56
Improvement	31	54.39	19	21.84	28	42.42	0	0.00	78	28.89
Female gender^c^	43^2,3,4^	75.44	27^1^	31.03	30^1,4^	45.45	14^1,,3^	23.33	114	42.22
Age^b^										
18–39 years	11^2,3,4^	19.3	0^1,3,4^	0.00	0^1,2,4^	0.00	3^1,2,3^	5.00	14	5.19
40–49 years	46	80.7	26	29.89	5	7.58	26	43.33	103	38.15
50 and over	0	0.00	61	70.11	61	92.42	31	51.67	153	56.67
Education^c^ (college or +)	19	33.33	28	32.18	19	28.79	22	36.67	88	32.59
Having children^c^	26	45.61	40	45.98	35^4^	53.03	19^3^	31.67	120	44.44
Need factors										
Common mental health disorders^c^	30^2^	52.63	20^1,3,4^	22.99	41^2,4^	62.12	24^2,3^	40.00	115	42.59
Severe mental health disorders^c^	18^2^	31.58	12^1,3^	13.79	27^2^	40.91	16	26.67	73	27.04
Substance use disorders^c^	22	38.6	21^3,4^	24.14	30^2^	45.45	29^2^	48.33	102	37.78
Perceived health^a^ (mean/SD)	7.12^2,3,4^	1.63	8.54^1,3,4^	1.12	5.68^1,2,4^	1.51	6.45^1,2,3^	1.84	7.08	1.87
Functional disability^a^ (mean/SD)	21.63^2,3^	6.17	14.82^1,3,4^	3.26	27.03^1,2,4^	6.89	21.71^2,3^	6.58	20.77	7.33
Enabling factors										
Frequency of service use										
Public primary care^d*^ (mean/SD)	4.68	5.89	5.24	11.34	4.52	4.81	3.70	5.60	4.60	7.82
Specialized ambulatory care^d*^ (mean/SD)	6.07^2,4^	14.21	1.84^1^	11.12	2.06^4^	7.96	0.48^1,3^	1.84	2.49	10.07
Community-based services^d*^ (mean/SD)	68.58	108.54	93.89	133.41	95.98	116.79	99.02	136.20	90.20	125.04
Acute care^d^ (mean/SD)	3.88^2^	13.86	1.25^1,3,4^	2.56	2.08^2^	3.27	3.52^2^	13.07	2.51	9.13
Satisfaction with services^a^ (mean/SD)	3.97^2^	0.85	4.39^1,3,4^	0.74	3.96^2^	0.81	3.83^2^	0.78	4.07	0.82

Group 1: “Young women in stable-PH or improved housing status with moderately high needs and specialized ambulatory care service use, with improved QOL over 12 months”

Group 2: “Middle-age to older men with stable housing status, few needs and low acute care service use, with most improvement in QOL over 12 months”

Group 3: “Older individuals residing in stable-PH or improved housing status with very high needs and reduced QOL over 12 months”

Group 4: “Men in stable-TH or worse housing status, with high SUD, using few specialized ambulatory care services and showing decline in QOL over 12 months”

*Public primary care (family doctor, walk-in medical clinic in LCSC or private clinic, other LCSC services); Specialized ambulatory care (outpatient hospital services other than hospitalizations and ED visits but including addiction rehabilitation centre); Community-based services (addiction treatment centre, support group, women’s centre, day centre, food bank, employment support programs and other organizations); Acute care: (Hospitalizations and ED visits)

Superscript numbers indicate significant differences at *p* < 0.05

*QOL* quality of life, *TH* temporary housing, *PH* permanent housing, *LCSC* local community service centre, *ED* emergency department

^a^T-test

^b^Fisher’s exact test

^c^Pearson’s chi-squared test

^d^Wilcoxon rank-sum test

Cluster analysis revealed four groups regarding change in QOL over 12 months, based on group comparisons for each variable (Table 2). Two groups (Groups 1 and 2) showed a positive change in QOL from T0 to T1. Group 1 represented 21% of the sample (n = 57/270), with average increase in QOL score of 4.6 (S.D. = 12.4), and Group 2 was the largest representing 32% of the sample (n = 87/270), with average improvement of 7.2 (S.D. = 10.8) in QOL score. The remaining two groups (Groups 3 and 4) demonstrated negative change in QOL over 12 months. Group 3 accounted for about 24% of the sample (n = 66/270) with a mean decreased QOL score of 3.2 (S.D. = 11.9) and for Group 4, including 22% of the sample, the QOL score declined of 1.9 (S.D. = 11.3). Differences in scores for change in QOL were significant for the direction of change, i.e. between groups with positive change (Groups 1 and 2) and negative change (Groups 3 and 4). However, the magnitude of change in QOL between Groups 1 and 2, or between Groups 3 and 4, was not significant. Table 3 shows group differences according to housing status and quality of life at T0 and T1, revealing that the QOL score for Group 2 at baseline was significantly higher than those of the other groups.

**Table 3:** Description of clusters according to housing status and quality of life at baseline (T0) and 12 months (T1)

	Group 1		Group 2		Group 3		Group 4		Total	
	n	%	n	%	n	%	n	%	n	%
Group size	57	21.11	87	32.22	66	24.44	60	22.22	270	100
Housing status										
Baseline										
Shelter	1	1.75	1	1.15	4	6.06	11	18.33	17	6.3
TH	32	56.14	43	49.43	29	43.94	30	50	134	49.63
PH	24	42.11	43	49.43	33	50	19	31.67	119	44.07
Time 1										
Shelter	0	0	2	2.3	0	0	20	33.33	22	8.15
TH	2	3.51	28	32.18	6	9.09	40	66.67	76	28.15
PH	55	96.49	57	65.52	60	90.91	0	0	172	63.7

Quality of life score	Mean	SD	Mean	SD	Mean	SD	Mean	SD	Mean	SD
Baseline^a^	68.68^2^	7.68	74.63^1,3,4^	9.90	69.11^2^	9.39	70.23^2^	10.45	71.05	9.76
Time 1	73.28	12.26	81.84	9.62	65.94	13.63	68.35	13.56	73.15	13.73

Superscript numbers indicate significant differences at *p* < 0.05

^a^T-test

In terms of QOL over 12 months, Group 2 showed the greatest improvement of the groups. Group 2 individuals were 40 years or older, predominantly male (69%), and most (71%) remained in stable TH or PH, rather than experiencing improvement in housing status (Groups 1 and 3) or deterioration (Group 4). Overall needs factors were lowest for Group 2, whose members were significantly less affected by common MHD than those in the three other groups, had less severe MHD compared with Groups 1 and 3, and less SUD compared with Groups 3 and 4. Individuals in Group 2 also had both significantly higher perceived health and lower functional disability scores compared with those in other groups. Concerning enabling factors, Group 2 individuals reported considerably lower use of acute care services and markedly higher service satisfaction compared with those in other groups. Moreover, Group 2 individuals used significantly fewer specialized ambulatory care services than Group 1. Group 2 was labeled: “Middle-age to older men with stable housing status, few needs and low acute care service use, with most improvement in QOL over 12 months”.

A positive change in QOL was also observed for Group 1, which mainly consisted of women (75%) and significantly younger (18–49 years old) than in other groups. Most Group 1 individuals were in stable-PH or had improved housing status, whereas none had experienced housing status deterioration, unlike in Groups 2 and 4. Prevalence of common and severe MHD in Group 1 was second highest of the groups, and significantly greater than in Group 2. However, Group 1 had a significantly higher perceived health score compared with the scores of groups with negative change in QOL (3 and 4), but lower compared with Group 2. Functional disability in Group 1 was markedly lower than in Group 3, but higher than Group 2. Regarding enabling factors, Group 1 individuals used specialized ambulatory care services significantly more than Group 2 individuals, but less than in Group 4. Group 1 was labeled: “Young women in stable-PH or improved housing status with moderately high needs and specialized ambulatory care service use, with improved QOL over 12 months”.

Group 4 participants had slightly negative change in QOL over 12 months, with significantly lower scores than Groups 1 and 2. Group 4 was predominantly male with a significantly higher proportion of individuals in stable-TH or with housing status deterioration than in the other groups. Group 4 participants had moderate needs, with fewer reporting common MHD, higher perceived health and lower disability scores compared with Group 3 participants, who also showed unfavourable change in QOL. However, Group 4 had the highest SUD prevalence, significantly greater than Group 2. Regarding enabling factors, Group 4 had the lowest mean frequency of specialized ambulatory care service use, considerably lower than in Groups 1 and 3. Group 4 was labeled: “Men in stable-TH or worse housing status, with high SUD, using few specialized ambulatory care services and showing decline in QOL over 12 months”*.*

Finally, Group 3 showed the most decline in QOL over 12 months compared with the other groups. As well, men and women were almost equally distributed, but most Group 3 individuals were 50 years old or more (92%). Most Group 3 participants were in stable-PH or improved housing status at T1, similar to those in Group 1. Needs factors were highest in Group 3, which also had the most individuals reporting common MHD, significantly higher than in Groups 2 and 4, or severe MHD, significantly higher than in Group 2. They also had the lowest perceived health and highest functional disability scores of all groups. Yet, overall, their scores on enabling factors were comparable to other groups, except for frequency of specialized ambulatory care service use, which was significantly higher than in Group 4, and satisfaction with services, which was significantly lower than in Group 2. Group 3 was labeled: “Older individuals residing in stable-PH or improved housing status with very high needs and reduced QOL over 12 months”*.*

## Discussion

This study developed a typology based on change in QOL over 12 months for individuals who were currently or formerly homeless and using different types of housing services in Quebec. The mean QOL scores in this sample at T0 (71.1) and T1 (73.2) were both lower than scores reported for the general population (77.5), according to a Quebec epidemiological study [19]. The lower QOL scores in our sample seemed logical, however, since people with financial difficulties generally have lower QOL, as is typical of homeless individuals [19]. The mean QOL score in this study was also relatively higher than that reported by O’Connell (56.8), which measured QOL among homeless individuals using another standardized instrument [9].

Just over half of study participants experienced improvement in QOL over 12 months. Four groups were identified through cluster analysis, two groups (Groups 1 and 2) revealing positive change in QOL over 12 months and two (Groups 3 and 4) showing negative change in QOL over the same period. Although comparisons with existing studies were difficult to make due to the heterogeneity among study samples and novelty of the dependent variable in this study, most groups showed some similarities with those identified in previous cluster analyses involving homeless individuals. For example, Bonin [29] identified a group of predominantly women making high use of services, like our Group 1. Group 3 in our study consisted of individuals residing in stable PH yet with multiple MHD and SUD, similar to groups described in previous studies [22, 23]. The profile for Group 3 was typical of a clientele targeted by Housing First programs [51, 52]. Profiles of homeless individuals mainly affected by SUD, similar to our Group 4, have been previously identified [18, 22]. However, to our knowledge, no studies have identified a profile like Group 2, i.e., individuals residing in stable housing who have low needs and make little use of services.

Group 2, with the highest mean change in QOL over 12 months, had rather a distinct profile on predisposing, needs, and enabling factors, compared with the other three groups. In terms of predisposing factors, baseline QOL was significantly highest, which implies that Group 2 individuals already perceived their QOL as relatively high even at the start of the study. In addition, the distribution of 12-month change in housing status was notably stable for Group 2, where most individuals remained in stable-TH or stable-PH rather than changing housing status. Their experience differed from that of individuals in the other groups, whose housing status either improved (Groups 1 and 3) or deteriorated (Group 4). The combined findings suggest that continuity and familiarity of living environment may provide a stronger sense of housing stability and satisfaction in the short-term, contributing to positive change in QOL [21, 39]. Moreover, concerning needs factors, Group 2 was the healthiest with the lowest prevalence of common MHD and functional disability, as well as the most positive perceived health compared with the other groups. The association between low severity of needs and higher QOL is strongly supported in previous studies [5, 53, 54]. Regarding enabling factors, Group 2 participants made least use of acute care services and were most satisfied with services, which may be explained by the fact of their having fewer needs.

Concerning needs factors, individuals in Groups 3 and 4, who experienced decline in QOL, rated their perceived health significantly lower than in Groups 1 and 2. Some studies have shown low perceived health to be a risk factor for poor life satisfaction in general [55], yet these findings are not as strongly correlated with QOL as with other clinical measures, such as MHD [7, 54].

Although Group 3 consisted of individuals residing in stable-PH or having improved housing status, this group showed a decrease in QOL over 12 months, which supports the notion that QOL may not be automatically associated with living in more favourable housing situations. One explanation may be that access to a PH program poses new and greater challenges for those individuals, as compared with living in TH or shelters [56]. For instance, the transition to PH may lead to a rupture with one’s previous social network and create feelings of loneliness and isolation [53, 57], which may, in turn, induce or worsen MH issues, including depression, SUD, and feelings of helplessness [58]. Group 3 was notably the group with the worst reported health status and highest prevalence of MHD and functional disability of all groups. Poor physical and MH conditions can result in functional disability, further contributing to the hardships faced by homeless individuals (e.g. unemployment) and jeopardizing overall well-being and QOL [15, 59]. The older age of Group 3 individuals may also explain their multiple health problems, functional disability rates and lower perceived health [32].

Group 4 individuals experienced considerable deterioration in housing status and reduced QOL over the 12 months. Although the reduction in QOL for Groups 3 and 4 was comparable, these findings for Group 4 participants were surprising, as they were younger, with lower functional disability and better perceived health. Finally, in terms of enabling factors, Group 4 used significantly fewer specialized care services than Groups 1 and 3. Lower service use (including addiction or other outpatient MH care services) among Group 4 individuals may have been due to the overrepresentation of men, especially those affected by SUD, as found in a previous study [60]. This seems to suggest that use of specialized care services may be mediated more by predisposing and needs factors than directly associated with QOL.

Group 1 showed greatest improvement in housing status at T1, and, while comparable to Group 3, Group 1 also improved in QOL. This suggests that achieving adequate housing is still important for increasing QOL in the homeless population [21]. Characteristics distinguishing Group 1 from the other groups were the overrepresentation of women and younger individuals. The lack of clear association between gender or age and QOL in homeless populations was reflected in a recent systematic review [13], which suggested that other factors may have a stronger influence over QOL. However, considering that women tend to attain more support from relatives and friends than men [61], it is also possible that this support contributed to increased QOL for this group. Social support is acknowledged as one of the strongest factors associated with QOL [19]. Moreover, homeless women were more likely than men to have a regular source of health care [62], especially specialized care in Group 1, which may also have contributed to their higher QOL.

Finally, this typology of change in QOL over 12 months revealed surprisingly few differences among enabling factors across the groups, specifically public primary care services. Previous US research on homelessness has described associations between increased public health service use, including primary care, and presence of MHD [63]. However, these increases may not play out in countries like Canada with fewer barriers to health care [60]. Moreover, the link between MHD and SUD with negative QOL [54, 64] suggests the need for public primary care use to boost QOL among homeless individuals, due to their high health needs. However, as previously reported, frequency of public service use in homeless populations may be more related to clinical variables than to QOL [63].

## Limitations

Some limitations to this study should be noted. First, as some domains of QOL seem to show greater improvement than others in homelessness [12, 16], analyzing multiple facets of QOL (e.g. personal relationships, autonomy) may have provided more domain-specific findings. Second, although structured interviews included validated scales and surveys, the data relied on participant self-report. Third, some key variables such as social support and having a family physician were not considered. Fourth, the sample may not be representative of the Quebec homeless population due to convenience sampling. Specifically, middle-age and older people (age 40 and over) were overrepresented relative to younger participants, and emergency shelter users were underrepresented. Fifth, study findings cannot be generalized beyond Quebec, especially to jurisdictions like the US without universal health care [65]. Finally, given the transience in housing for this population, especially among individuals in shelters or TH, future studies could be conducted with additional follow-up points within the 12-month period to more closely capture changes in QOL with respect to changes in housing status.

## Conclusions

This study was the first to develop a typology based on change in QOL over a 12-month period among homeless individuals using three types of housing services based on an adapted Gelberg–Andersen model. Several novel or rarely examined variables related to QOL were considered, including change in housing status, perceived health, frequency of public primary care, specialized ambulatory care and community-based service use, as well as service satisfaction. Cluster analysis identified four distinct groups, two revealing positive change and two showing negative change in QOL over 12 months. Results suggested that stable housing status may influence QOL more strongly over 12 months than improvement in housing status. Moreover, positive change in QOL was mainly associated with fewer needs variables. Some recommendations related to the different profiles of service users may support efforts to improve QOL. For individuals resembling Group 3, with the most negative change in QOL, prioritizing assertive community may promote increased health service use in view of their multiple health problems. Concerning individuals like Group 4, who also experienced decline in QOL, harm reduction may be promising to decrease SUD, which likely contributed to deterioration in housing status, health, and QOL. Finally, for the two other groups (Groups 1 and 2) that showed improvements in QOL, achievement or maintenance of housing stability, combined with service use corresponding to their needs may sustain or improve levels of perceived QOL.

## Data Availability

In accordance with the applicable ethics regulations in the province of Quebec, the informed consent form must specifically inform the participants of the possibility of research data sharing with third parties, as well as the limitations and safety measures associated with such data sharing. Since the consent form approved by the ethics committee of a MH university institute signed by participants for this research project did not specifically provide for such data sharing, the principal investigator is responsible to the study participants for keeping these data confidential.

## Abbreviations

ED: Emergency department
LCSC: Local community service centre
MH: Mental health
MHD: Mental health disorders
PH: Permanent housing
QOL: Quality of life
SUD: Substance use disorder
TH: Temporary housing
